# Occupational trajectories of working conditions in Sweden: Development trends in the workforce, 1997–2015

**DOI:** 10.5271/sjweh.3955

**Published:** 2021-06-29

**Authors:** Linda Corin, Anders Pousette, Tomas Berglund, Lotta Dellve, Gunnel Hensing, Lisa Björk

**Affiliations:** Institute of Stress Medicine, Region Västra Götaland, Gothenburg, Sweden; Department of Sociology and Work Science, University of Gothenburg, Gothenburg, Sweden; Department of Psychology, University of Gothenburg, Gothenburg, Sweden; School of Public Health and Community Medicine, Institute of Medicine, University of Gothenburg, Gothenburg, Sweden

**Keywords:** job demand, job resource, macro trend, meso trend, official statistic, polarization, work environment

## Abstract

**Objective:**

This study aimed to explore the development of working conditions within and between occupations in the Swedish labor market from 1997 to 2015 and whether any polarization in working conditions concurrently occurred between occupations.

**Methods:**

Cross-sectional data from ten waves of the Swedish Work Environment Surveys (1997–2015) were used and an aggregated occupational-level dataset was created using the Swedish Standard Classification of Occupations. To capture the patterns of change in working conditions over time (ie, growth), growth curve modeling was used to identify the starting points for 89 occupations (intercepts) as well as both the shape (functional form) and rate of growth (slope) over time.

**Results:**

The Swedish labor market was stable overall, with some small, mainly positive, changes in job demands and resources. Different occupations developed in divergent directions, but there was no evidence of polarization.

**Conclusions:**

The findings indicate that macro-level stability can hide highly heterogeneous patterns of change among different occupational groups. This type of analysis, taking context into account, could be valuable for decision makers intending to improve the work environment.

The work environment is very important for employee health and productivity. Thanks to decades of extensive occupational health and safety research, the physical and psychosocial working conditions that constitute risks and resources are well known in Europe (1, 2). These conditions can theoretically be sorted into demands and resources and applied in the job demand–resources (JD–R) model (3), a well-recognized framework for capturing working conditions. Many studies relate job demands and resources to health and motivational outcomes, using both the JD–R and other preceding models (for an overview, see for example 4, 5). However, it is less common to use the JD–R model in a macro-level (for example, labor market) setting to investigate the broad development of working conditions in different occupations over time. With a focus on patterns and directions in the development of important job demands and resources in different occupations, this study sets out to examine working conditions within and between occupations from 1997 to 2015.

In the 1970s and 1980s, Sweden took important steps in improving the work environment, for example, implementing regulations governing working conditions, passing legislation regarding the occupational health service, and starting several national institutes of occupational safety and health. The economic crisis of the early 1990s changed the labor market and working conditions in many respects (6). Swedish national financial policy changed its main priority from ensuring high employment to combating inflation and, at the same time, the conditions for international trade changed. The unemployment rates rose rapidly and almost tripled in size. Discussions on making the private and especially public sector more efficient intensified. Major restructuring of the private and public sectors followed, with eg, slimmed-down organizations, creating significant turbulence. In addition, the legal obligations of the occupational health service were removed. Taken together, a series of extensive changes on the labor market, like other similar countries implemented over a long period, were concentrated in a very short time in Sweden. Many employees consequently experienced deteriorated work environments (6–13). Guided by Sweden’s official work environment statistics, the greatest deteriorations were found in psychosocial working conditions (6, 14), with general work intensification and substantial increases in job demands. The work intensification was later on accompanied by decreases in job control (6, 9, 15). The number of Swedish employees in high-strain work thus increased during the 1990s.Those working within the public sector were hit the hardest. In Swedish healthcare, for example, the proportion who answered that they had “too high job demands” increased by as much as 25% between 1991–1999, and job control decreased by >10% between 1995–1999 (6).

By following the same official Swedish work environment statistics – but over a longer period – Gellerstedt (16) found both positive and negative developments for manual workers between 1991–2014. In line with this, Cerdas et al (17) demonstrated that job demands, decision authority, and social support developed in different directions between 1991–2013. For example, a trend toward increasing demands and decreasing decision authority was more salient in female-dominated sectors. These findings indicate that overall macro trends might conceal different meso-level trends and that occupations might develop in divergent directions.

Other indications of concealed work environment heterogeneity are the recent findings of polarized occupational structure (18, 19). Polarization refers to a pattern of occupational change in which employees in both high- and low-skilled occupations are growing in numbers, while medium-skilled employment is being hollowed out. Technological change, in particular digitalization, is believed to be the main cause of this due to its potential to replace routine work tasks. Such occupations are found in the middle of the skill structure (for example, assemblers and office clerks). Non-routine jobs are mainly high-skilled, with digital technology instead tending to complement the work and increase productivity. However, there is also a tail of low-skilled manual jobs with non-routine characteristics (for example, waiters) that are not easy to replace with digital devices. This tail is tending to grow in relative numbers.

In the Swedish case, some scholars have found that the upgrading that previously characterizing the labor market has given way to polarization in recent decades (20–22). Upgrading refers to a process in which low-skilled and often low-quality jobs are replaced with more and better high-skilled jobs (23). However, in recent decades, the low-skilled tail of the occupational structure has not continued to shrink; instead, these the number of these jobs has increased. Changes in the occupational structure have been measured using wages as a proxy for skills. This approach has been criticized, as wages do not straightforwardly mirror skill requirements (24). Using individuals’ own assessments of job requirements, Tåhlin (24) found no polarization, but rather continuing upgrading on the Swedish labor market. Oesch & Piccitto (25) expanded the analysis to encompass measures of job quality besides wages – such as educational level, prestige, and job satisfaction – and did not find any evidence of polarization.

The direction of changes in the occupational structure are important since polarization may have consequences for work environments. Kalleberg (26) argued that the polarization process entails a divide between “good” and “bad” jobs, suggesting a trend towards greater inequality, while Peugny (27) showed that precarious employment conditions have become more common in the low-skilled segment over the last 20 years.

In this study, we take a comprehensive approach to the question of how working conditions have evolved within and between occupations in recent decades, focusing on central dimensions of the JD–R model. Is the Swedish occupational structure moving in the direction of polarization, or has there been a positive trend of upgrading with a decrease in unsatisfying and hazardous working conditions?

Our aim was to explore the development of working conditions among occupations on the Swedish labor market during the 1997–2015 period. Specifically, this study investigates: (i) the overall trends in both physical and psychosocial job demands and resources, ie, the macro trends; (ii) the divergent trends in the development of working conditions between occupational groups, ie, the meso trends; and (iii) whether the variation between occupations has increased in a polarized manner over time.

## Method

### Study population and data collection

The Swedish Work Environment Survey (SWES), conducted biannually since 1989 by the Swedish Work Environment Authority (SWEA) and Statistics Sweden (SCB), consists of a random, stratified representative subsample of gainfully employed Swedes (~4–4,5 million individuals during the study period). The gainfully employed includes all individuals aged 16–64 years who have worked for ≥1 hour during the measurement week in salaried work, as self-employed, or in a family business. Hence, the sample also includes those who take on shorter assignments and thus have atypical employments.

The SWES subsample is drawn from the regular Labor Force Survey (LFS) conducted by SCB and varying between approximately 10 000–15 000 individuals (depending on the number of gainfully employed in a given year). The LFS is in turn drawn from the Register of the Total Population (RTB) as selection frame and consists of a representative sample of the whole Swedish population stratified by county, sex, and age group. The LFS is conducted by means of telephone interviews, and those who are chosen to participate in the SWES are asked additional questions during these interviews and to complete a supplementary postal or web questionnaire. The survey has been conducted using a similar methodology from its launch in 1989. In total, approximately 130 questions about physical and psychosocial working conditions are asked.

Dropout occurs at each step, first in the LFS and then in SWES, due to, for example, problems related to health, language, and available time. Prior to 2002, the dropout rate in the LFS was low and relatively stable. However, since then, the dropout has steadily increased, with the greatest increases occurring since 2009 and especially since 2013. Therefore, in 2015, LFS dropout was thoroughly assessed for 2002–2014 (28). The analysis showed that dropout has consistently been slightly higher among men, although this difference between the sexes has diminished over time. The dropout for the foreign-born and those living in densely populated areas has consistently been higher during this period. The dropout between different age categories has increased over time, with the highest dropout among 15–24-year-olds. Similarly, dropout has increased more among those with a lower educational level. Taken together, dropout has roughly doubled in the LFS since 2002. However, there is no information in the LFS on how large the dropout rate is for the subgroups employed versus not employed (29).Therefore, SWEA states that there are no prerequisites with reasonable certainty estimating how large the dropout rate was among those employed between 1997–2015 and, thus, how large the error can be assumed to be in SWES (30).

Even so, we know that the number of participants in the SWES has decreased over time (table 1); an attempt to conduct a more solid dropout analysis of SWES was made in 1999 (30). Similar to the dropout analysis of the LFS, the analysis revealed lower response rates among men, the young, and employees with low education and foreign background. Participation was also lower among those with low income, contract or part-time employment or with own businesses. Still, the response rate in SWES remains relatively high (table 1) and constitutes the best available official statistics and data source in Sweden concerning working conditions over time.

**Table 1:** Total n in the sample per year, crude number of responses per year and response %. Total number of observations in the final sample per year, after exclusion of occupations with few respondents.

Year	Total N	Crude N	Response %	Final N
1997	14 053	12 886	92	12 720
1999	14 234	12 535	88	12 395
2001	14 402	12 878	89	12 721
2003	14 317	12 355	86	12 203
2005	15 562	13 538	87	13 357
2007	12 118	10 671	88	10 530
2009	11 045	9152	83	9058
2011	15 553	12 367	80	12 219
2013	9810	8110	83	8009
2015	8895	7336	82	7228
Total	129 989	111 828	86	110 440

### Aggregation to occupational level

We created a dataset of longitudinal occupational data for the Swedish labor market between 1997–2015 (in some cases 2013) using the Swedish Standard Classification of Occupations (SSYK). Similar to international standard classification systems (for example, ISCO by ILO), SSYK covers type of work and qualifications required. A new version of SSYK was introduced in SWES in 2012 and, by using translation keys between the older SSYK96 and the current SSYK12, ten survey rounds of the SWES could be created for this study. Observations from these rounds were compiled, generating a dataset with data for the years 1997–2015 (N=111 828 individual observations).

The three-digit level of SSYK comprises 113 occupations. However, 21 small occupational groups (eg, senior officials of special interest organizations, models, religious professionals, ships deck’s crew and street vendors) with few observations (N<15 for more than 50% of survey rounds) were excluded (N=1388), and four occupations in the process industry were merged into one (see supplementary material, www.sjweh.fi/show_abstract.php?abstract_id=3955, table S1). Thus, observations from 89 occupations provided the basis for the final dataset, containing 110 440 individual observations (table 1). The data were aggregated to the occupational level, rendering a set of longitudinal data with ten waves of 89 occupations for the years 1997–2015.

### Measures

Changes were made to SWES in 1995, 2005, and 2013, resulting in the rewording of some questions (31). Even so, 43 working conditions could be compared over the full study period (1997–2015) and an additional 5 over almost the full study period (1997–2013). Of these 48 dimensions, 24 were chosen to gain broad representation of physical and psychosocial working conditions. Questions with yes/no response alternatives and questions capturing very specific physical demands were not included. To facilitate interpretation, these 24 individual dimensions were categorized into four job resources and four job demands (table 2) using the JD–R model as a conceptual framework (32). To facilitate interpretation, all dimensions were normatively coded so that a high value implies a favorable work condition, thus a positive regression estimate of the slope coefficient implies improvement over time.

**Table 2:** Dimension description. *Italics: The Swedish Work Environment Authorities official translation from Swedish to English have been used for all the questions included in the postal/web questionnaire. The questions in italics represents the questions asked by means of telephone interviews. Since no official translation of these questions are available. The authors did the translations.* [R=response alternatives have been reversed compared with the original scale.]

Domain	Dimension	Dimension formulation	Response scale in present study
**Job resources**			
Influence	Autonomy ^a^	*Do you feel that you have too little or too much influence in your work?*	*1 = too little influence, fully agree, 2 = too little influence, partly agree, 3 = neither/nor, 4 = too much influence, partly agree,*
*5 = too much influence, completely agree*
	Decision authority: pace	Do you have the opportunity to determine your work pace?	1 = no, not at all, 2 = about 1/10 of the time, 3 = about 1/4 of the time, 4 = half the time, 5 = about 3/4 of the time, 6 = nearly all the time (R)
	Decision authority: when	Are you able to determine when various work duties are to be carried out (for example, by choosing to work a bit faster on some days and taking it easier on other days)?	1 = no, not at all, 2 = mostly not, 3 = mostly, 4 = always (R) ^b^
	Decision authority: what, how	Do you participate in decisions on the arrangement of your work (e.g., what is to be done, how to do it, or who will work with you)?	1 = no, not at all, 2 = mostly not, 3 = mostly, 4 = always (R) ^b^
	Unbound and free	*Do you feel that your work is bound and* *unfree or that it is unbound and free?*	*1 = bound and unfree, fully agree, 2 = bound and unfree,* *partially agree, 3 = neither/nor, 4 = unbound and free, partially agree, 5 = unbound and free, fully agree*
Social support	Support from colleagues	Are you able to get support and encouragement from colleagues when work feels difficult?	1 = no, not at all, 2 = mostly not, 3 = mostly, 4 = always (R) ^b^
	Appreciation	Do other people show appreciation for things you do (e.g., colleagues, patients, customers, clients, passengers, and students)?	1 = not at all/rarely the last 3 months, 2 = a couple of days per month (1 day of 10), 3 = one day per week (1 day of 5), 4 = a couple of days per week (1 day of 2), 5 = every day (R) ^b^
	Supervisor support ^a^	Are you able to get support and encouragement from supervisors when work feels difficult?	1 = always, 2 = mostly, 3 = mostly not, 4 = no, not at all
	Supervisor appreciation ^a^	Does your supervisor show appreciation for things you do?	1 = every day, 2 = a couple of days per week (1 day of 2), 3 = one day per week (1 day of 5), 4 = a couple of days per month (1 day of 10), 5 = not at all/rarely the last 3 months
Recovery	Pause opportunities	Can you take short breaks at virtually any time?	1 = no, not at all, 2 = about 1/10 of the time, 3 = about 1/4 of the time, 4 = half the time, 5 = about 3/4 of the time, 6 = nearly all the time (R)
Meaningfulness	Meaningfulness	*Do you feel that much of your work is meaningless or meaningful?*	*1 = very meaningless work, fully agree, 2 = very meaningless work, partly agree, 3 = neither/nor, 4 = very meaningful work, partly agree, 5 = very meaningful work, completely agree*
**Job demands**			
Cognitive	Difficulty of work tasks	*Do you feel that you have too difficult or too simple tasks in your work?*	*1 = far too difficult, fully agree, 2 = far too difficult, partly agree, 3 = neither/nor, 4 = far too simple, partly agree, 5 = far too simple, completely agree*
	Monotony	*Do you feel that your work is monotonous or varied?*	*1 = monotonous work, fully agree, 2 = monotonous work, partly agree, 3 = neither/nor, 4 = varied work, partly agree, 5 = varied work, completely agree*
	Concentration	Does the work require your full attention and concentration?	1 = no, not at all, 2 = about 1/10 of the time, 3 = about 1/4 of the time, 4 = half the time, 5 = about 3/4 of the time, 6 = nearly all the time (R)
	Psychological pressure	*Do you find your work mentally stressful* *or calm and pleasant?*	*1 = mentally stressful work, fully agree, 2 = mentally stressful work, partly agree, 3 = neither/nor, 4 = mentally easy work, partly agree, 5 = mentally easy work, completely agree*
Quantitative	Workload	*Do you feel that you have far too much* *or too little to do in your work?*	*1 = far too much to do, fully agree, 2 = far too much to do, partly agree, 3 = neither/nor, 4 = far too little to do, partly agree, 5 = far too little to do, completely agree*
	Work–leisure spillover	Do you find that you cannot stop thinking about work when you are free?	1 = every day, 2 = a couple of days per week (1 day of 2), 3 = one day per week (1 day of 5), 4 = a couple of days per month (1 day of 10), 5 = not at all/rarely the last 3 months
	Time pressure	Is your work so stressful that you do not have time to talk or even think about anything other than work?	1 = nearly all the time, 2 = about 3/4 of the time, 3 = half the time, 4 = about 1/4 of the time, 5 = about 1/10 of the time, 6 = no, not at all
	Overtime	Do you have so much work that you must miss lunch, work late, or take work home?	1 = every day, 2 = a couple of days per week (1 day of 2), 3 = one day per week (1 day of 5), 4 = a couple of days per month (1 day of 10), 5 = not at all/rarely the last 3 months
Emotional	Emotionally demanding contacts	Do you sometimes come in close contact through your work with severely ill people or people with severe problems?	1 = every day, 2 = a couple of days per week (1 day of 2), 3 = one day per week (1 day of 5), 4 = a couple of days per month (1 day of 10), 5 = not at all/rarely the last 3 months
	Violence and threats	Are you exposed to violence or threats of violence in your work?	1 = every day, 2 = a couple of days per week (1 day of 2), 3 = one day per week (1 day of 5), 4 = a couple of days per month (1 day of 10), 5 = a few times in the last 3 months, 6 = a few times in the last 12 months, 7 = not at all in the last 12 months
Physical	Work postures	Do you feel that you have strenuous or comfortable working positions in your work?	1 = strenuous, fully agree, 2 = strenuous, partly agree, 3 = neither/nor, 4 = comfortable, partly agree, 5 = comfortable, completely agree
	Bend and twist	Do you bend or twist yourself in your work in the same way repeatedly in an hour, for several hours during the same day?	1 = every day, 2 = a couple of days per week (1 day of 2), 3 = one day per week (1 day of 5), 4 = a couple of days per month (1 day of 10), 5 = not at all/rarely the last 3 months
	Physical workload	Do you feel that you have strenuous heavy work or that it is physically very easy?	1 = physically strenuous work, fully agree, 2 = physically strenuous work, partly agree, 3 = neither/nor, 4 = physically easy work, partly agree, 5 = physically easy work, completely agree

### Analytic strategy

To capture patterns of occupational change in working conditions over time (ie, growth), growth curve modeling (GCM), a subset of hierarchal linear modeling specifically designed for longitudinal analyses, was used. GCM enables us to analyze the central tendency and variation in initial status (or starting point) of the growth (in the analyzed time frame) of occupations (intercepts) as well as both the shape (functional form) and rate (average slope and variation in slope) of growth over time. By using GCM, we consider the possibility that different occupations might have different intercepts defining their growth trajectories as well as different slopes. Before the GCM analyzes described below, the residuals were analyzed. The assumptions of constant variance, normality and linearity of the residuals were met for all of domains except Emotional demands, were the dimensions “Emotionally demanding contacts” and “Violence and threats” were found to be highly skewed, with most occupations not experience such demands at work.

### Assessing macro and meso trends: specifying and fitting the growth curve model

The analyses were performed using the mixed models unit in SPSS version 24 (IBM Corp, Armonk, NY, USA). Time (ie, intra-occupational growth over time) was set as Level 1 of the hierarchy. Observations over time were nested within occupations (Level 2) constituting the inter-occupational growth. The final sample consisted of ten time waves at Level 1 and 89 occupations at Level 2. The maximum likelihood (ML) method was used to estimate the statistical parameters in order to permit likelihood ratio testing. Both linear and nonlinear changes over time were examined. By using the “unstructured” covariance type, estimation of both the variance and covariance of the random effects was allowed (33).

A series of analytical steps was performed for each dimension. In the first step, an unconditional mean model (Model I) was estimated to serve as a baseline model for examining occupational variation in the work condition at hand, without regard to time. In Model I, (i) the mean of the outcome dimension and (ii) the amount of outcome variation existing within and between occupations were assessed. In the second step, an unconditional fixed linear growth curve model (Model II) was estimated to capture the linear development over time (ie, linear macro trends, the test of the first research question). Time was scaled as years divided by ten, implying that the slope coefficient should be interpreted as the change over a 10-year period. In the third step, an unconditional random linear growth curve model (Model III) was estimated to capture the variation in occupational development trends over time (ie, the meso trends, the test of the second research question). In the fourth step (Model IV), quadratic and cubic growth curve models were estimated to identify parabolic or S-shaped (ie, nonlinear) growth curves (the test of functional form of the macro trends, related to the first research question) (34).

### Calculation of effect sizes

The effect size for overall change in occupations over a 10-year period was calculated using the following procedure:


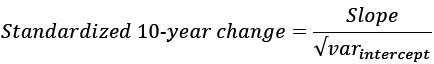


the fixed estimate of the slope coefficient was divided by the standard deviation of the intercept (ie, the variation between occupations) according to the following formula:


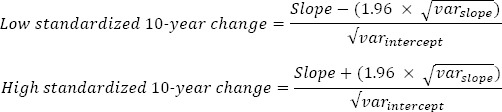


The effect size for trajectories of occupations was calculated as the range of change for 95% of the occupations over a 10-year period using the following formulas:

All calculations of effect sizes were based on Model III, with the linear slope only.

### Polarization analysis

To detect a trend towards polarization, the covariance between the intercepts and the slope in the multilevel models for each dimension was estimated. A positive covariance indicates a “fanning out” pattern of the trajectories, and thus greater differences between the occupations over time. A positive covariance would thus give support for polarization between the occupations.

## Results

Table 3 shows the lowest and highest observed means and standard deviations (SD) (based on occupational-level data) between 1997–2015 for the 24 included working conditions.

**Table 3:** Descriptives: lowest and highest means and standard deviations (SD) among the ten data points, 1997–2015, based on the aggregated (occupational level) dataset. Interclass correlation (ICC) (1,1) for 1997 and 2015 based on individual-level data with occupation.

Domain	Dimension	Mean		SD		ICC	
		
		Min	Max	Min	Max	1997	2015
**Job resources**							
Influence	Decision authority; pace	4.14	4.32	0.62	0.71	0.12	0.12
	Decision authority; when	2.51	2.63	0.38	0.45	0.18	0.20
	Decision authority; what, how	2.87	2.95	0.37	0.41	0.17	0.16
	Unbound and free	3.42	3.48	0.38	0.44	0.11	0.10
Social support	Social support from colleagues	3.03	3.10	0.20	0.31	0.06	0.05
	Appreciation	2.85	3.02	0.36	0.41	0.08	0.05
Recovery	Pause opportunities ^a^	3.99	4.17	0.66	0.79	0.14	0.14
Meaningfulness	Meaningfulness	3.87	3.95	0.37	0.43	0.15	0.08
**Job demands**							
Cognitive	Difficulty of work tasks	3.01	3.11	0.16	0.19	0.06	0.02
	Monotony	3.44	3.58	0.54	0.63	0.21	0.15
	Concentration	4.88	5.01	0.35	0.44	0.07	0.06
	Psychological pressure ^a^	2.68	2.94	0.33	0.39	0.13	0.07
Quantitative	Workload	2.22	2.45	0.21	0.28	0.07	0.05
	Work-leisure spillover	3.41	3.63	0.49	0.60	0.16	0.11
	Time pressure ^a^	3.93	4.12	0.38	0.51	0.08	0.05
	Overtime ^a^	3.74	3.95	0.44	0.54	0.16	0.13
Emotional	Emotionally demanding contacts	4.15	4.28	0.86	0.90	0.39	0.35
	Violence and threats	6.75	6.79	0.31	0.41	0.16	0.17
Physical	Work postures	2.93	3.12	0.49	0.60	0.23	0.21
	Bend and twist	3.30	3.71	0.81	0.91	0.19	0.22
	Physical workload ^a^	3.41	3.49	0.77	0.83	0.36	0.34

a The time-series was disrupted in 2013 due to rewording of the SWES (2015), resulting in a shortened time series (1997–2013).

Table 3 also includes calculations of the intraclass correlation coefficient [ICC (1, 1)] based on individual-level data and with occupations as the grouping variable, ie, the amount of variance attributable to the occupational (meso) level (35). Roughly 2–39% of the variance in the 24 studied working conditions could be attributed to the occupational meso level. The remaining variance may thus be explained by aspects associated with workplace, employee-specific characteristics, and measurement error. Based on low occupational-level variance, ie, ICC (1,1) values <5%, three dimensions were omitted from the final analysis: supervisor support ICC (1,1)=3%, supervisor appreciation ICC (1,1) =3%, and autonomy ICC (1,1)=4%. Thus, a total of 21 working conditions with enough variance attributable to the occupational level was used for the main analysis.

The results of the growth curve models estimating the occupational trajectories of working conditions are presented in table 4 (fixed parameters) and table 5 (random parameters).

**Table 4:** Growth curve models: parameter estimates for fixed effects, representing the average effect for all occupations (macro-level effects). [SD=standard deviation.]

Domain	Dimension	Intercept		Linear slope			Quadratic slope		Cubic slope	
			
		Estimate	Error	Estimate	Error	SD10-y change ^a^	Estimate	Error	Estimate	Error
**Job resources**										
Influence	Decision authority;pace ^b^	**4.261**	0.068	–0.048	0.025	–0.079				
	Decision authority; when ^c^	**2.522**	0.044	**0.191**	0.079	0.016	**–0.250**	0.105	**0.088**	0.038
	Decision authority; what, how ^b^	**2.895**	0.041	0.012	0.012	0.032				
	Unbound and free ^b^	**3.450**	0.041	0.001	0.016	0.001				
Social support	Support from colleagues ^b^	**3.080**	0.019	–0.013	0.011	–0.085				
	Appreciation ^c^	**2.863**	0.042	0.186	0.103	**0.156**	**–0.342**	0.136	**0.161**	0.050
Recovery	Pause opportunities ^b, d^	**4.072**	0.074	–0.032	0.027	–0.047				
Meaningfulness	Meaningfulness ^b^	**3.904**	0.045	0.008	0.014	0.020				
**Job demands**										
Cognitive	Difficulty of work tasks ^b^	**3.039**	0.017	**0.027**	0.007	0.189				
	Monotony ^b^	**3.518**	0.064	0.003	0.016	0.004				
	Concentration ^b^	**4.968**	0.037	–0.031	0.019	–0.099				
	Psychological pressure ^c, d^	**2.693**	0.042	–0.122	0.099	0.486	**0.524**	0.150	**–0.227**	0.061
Quantitative	Workload ^c^	**2.252**	0.025	0.049	0.074	**0.473**	0.168	0.098	**–0.086**	0.036
	Work–leisure spillover ^b^	**3.540**	0.057	–0.026	0.019	–0.050				
	Time pressure ^c, d^	**3.916**	0.047	**0.502**	0.146	0.048	**–0.631**	0.220	**0.220**	0.090
	Overtime ^c, d^	**3.714**	0.053	**0.059**	0.053	0.030	**–0.688**	0.175	**0.218**	0.072
Emotional	Emotionally demanding contacts** ^b^	**4.291**	0.092	**–0.065**	0.017	**–0.075**				
	Violence and threats ^b, e^	**6.774**	0.040	0.005	0.010	0.012				
Physical	Work postures ^c^	**2.989**	0.059	–0.182	0.094	**0.164**	**0.282**	0.126	–0.079	0.046
	Bend and twist ^c^	**3.542**	0.093	**–0.848**	0.135	**0.195**	**1.100**	0.180	**–0.321**	0.066
	Physical workload ^b, d^	**3.437**	0.085	0.014	0.015	0.017				

a Linear standardized 10-year change (standardized based on variation between occupations), calculated using Model III (linear change only). Bold numbers indicate significant fixed slopes in Model III; **bold numbers indicate P-value <0.05.**

b Fixed parameters were estimated using Model III (linear macro trend).

c Fixed parameters were estimated using Model IV (nonlinear macro trend).

d The time series was disrupted in 2013 due to rewording of the SWES (2015), resulting in a shortened time series (1997–2013).

e The dimensions “Emotionally demanding contacts” and “Violence and threats” are highly skewed, with most professions reporting close to the maximum in the scale and thus not experiencing such demands at work.

**Table 5:** Growth curve models: parameter estimates for random effects, representing variation between occupations in intercept and slope (mesolevel effects). [SD=standard deviation.]

Domain	Dimension	Intercept variance		Slope variance		Slope effect size		Residual variance		Covariance intercept- slope	
				
		Estimate	Error	Estimate	Error	SD 10-y change (low) ^a^	SD 10-y change (high) ^a^	Estimate	Error	Estimate	Error
**Job resources**											
Influence	Decision authority; pace ^b^	**0.373**	0.061	**0.024**	0.008	**–0.578**	**0.420**	**0.097**	0.005	**–0.037**	0.017
	Decision authority; when ^c^	**0.150**	0.024	**0.005**	0.002	**–0.335**	**0.367**	**0.026**	0.001	–0.006	0.005
	Decision authority;what, how ^b^	**0.141**	0.023	**0.004**	0.002	**–0.315**	**0.379**	**0.027**	0.001	**–0.010**	0.005
	Unbound and free ^b^	0.135	0.022	**0.013**	0.004	**–0.597**	**0.600**	**0.037**	0.002	**–0.014**	0.007
Social support	Support from colleagues ^b^	**0.022**	0.005	0.002	0.002	–0.723	0.553	**0.029**	0.002	0.003	0.002
	Appreciation ^c^	**0.124**	0.021	**0.011**	0.004	**–0.431**	**0.744**	**0.043**	0.002	**–0.151**	0.007
Recovery	Pause opportunities ^b, d^	**0.455**	0.073	**0.029**	0.010	**–0.540**	**0.455**	**0.089**	0.005	-0.033	0.020
Meaningfulness	Meaningfulness ^b^	**0.167**	0.027	**0.007**	0.003	**–0.370**	**0.409**	**0.036**	0.002	**–0.024**	0.007
**Job demands**										
Cognitive	Difficulty of work tasks ^b^	**0.021**	0.004	0.001	0.001	–0.129	0.507	**0.013**	0.001	–0.002	0.001
	Monotony ^b^	**0.352**	0.055	0.006	0.003	–0.255	0.263	**0.052**	0.003	**–0.037**	0.011
	Concentration ^b^	**0.100**	0.018	**0.011**	0.005	**–0.900**	**0.659**	**0.067**	0.004	**–0.173**	0.008
	Psychological pressure ^c, d^	**0.127**	0.021	**0.008**	0.003	**0.014**	**0.957**	**0.030**	0.002	**–0.024**	0.007
Quantitative	Workload ^c^	**0.039**	0.007	0.002	0.001	0.091	0.855	**0.023**	0.001	–0.001	0.002
	Work–leisure spillover ^b^	**0.270**	0.044	**0.012**	0.005	**–0.471**	**0.371**	**0.068**	0.004	**–0.035**	0.012
	Time pressure ^c, d^	**0.138**	0.024	**0.015**	0.007	**–0.581**	**0.677**	**0.066**	0.004	–0.012	0.010
	Overtime ^c, d^	**0.214**	0.034	0.006	0.004	–0.259	0.318	0.419	0.002	–0.016	0.008
Emotional	Emotionally demanding contacts ^b, e^	**0.741**	0.113	**0.012**	0.004	**–0.326**	**0.175**	**0.041**	0.002	–0.019	0.015
	Violence and threats ^b, e^	**0.135**	0.021	**0.004**	0.001	**–0.311**	**0.336**	**0.018**	0.001	**–0.011**	0.004
Physical	Work postures ^c^	**0.283**	0.044	**0.006**	0.003	**–0.113**	**0.441**	**0.037**	0.002	**–0.020**	0.008
	Bend and twist ^c^	**0.711**	0.110	**0.011**	0.005	**–0.013**	**0.403**	**0.075**	0.004	–0.032	0.018
	Physical workload* ^b^	**0.625**	0.096	0.006	0.003	–0.170	0.204	**0.034**	0.002	-0.019	0.012

a All slope effect sizes were estimated using Model III. **Bold numbers indicate P-value < 0.05.**

b Variance and covariance parameters were estimated using Model III (linear macro trend).

c Variance and covariance parameters were estimated using Model IV (nonlinear macro trend).

d The time series was disrupted in 2013 due to rewording of the SWES (2015), resulting in a shortened time series (1997–2013).

e The dimensions “Emotionally demanding contacts” and “Violence and threats” are highly skewed, with most professions reporting close to the maximum in the scale and thus not experiencing such demands at work.

### Macro trends in development of job demands and resources

**Figure 1:**
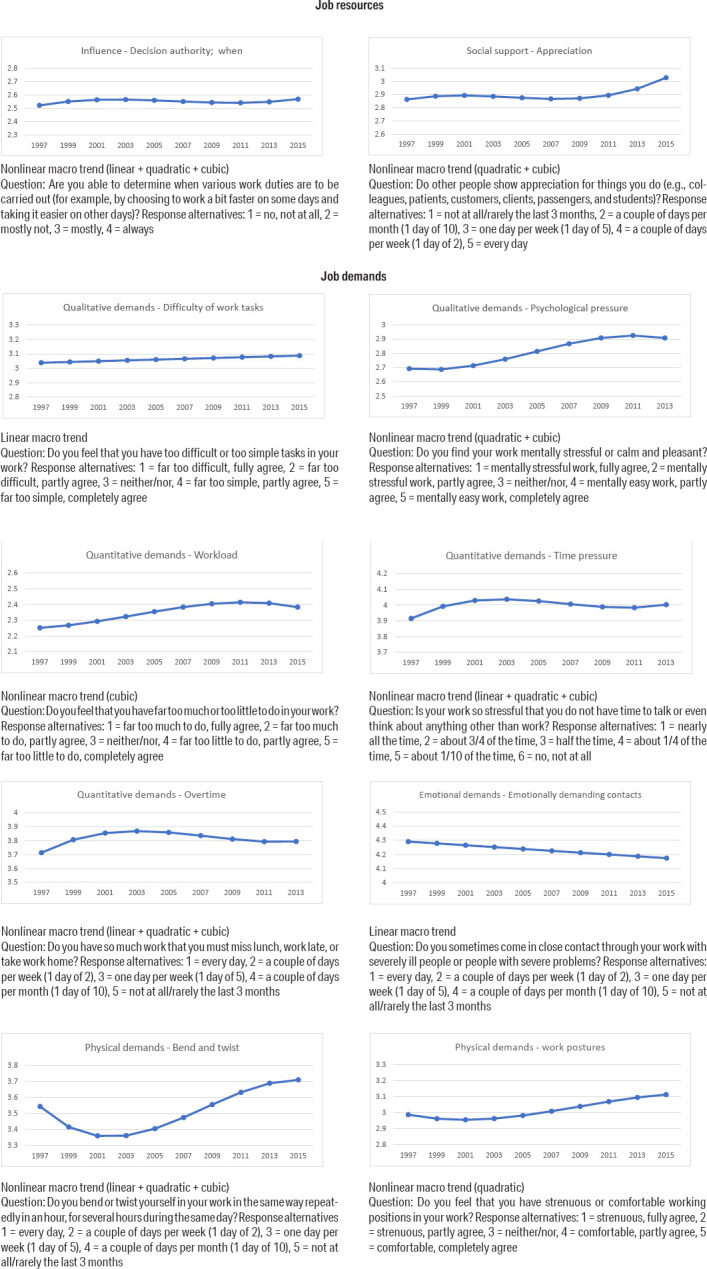
Nonlinear and linear macro trends; trajectories based on estimated parameters from Model IV and III respectively. Note that all dimensions have been coded so that a high value implies a favourable development in the working condition at hand.

Of the 21 working conditions, 10 displayed an overall macro-level development trend, shown as a significant linear, quadratic and/or cubic slope coefficient in Table 4. Two of the job demands (difficulty of work tasks and emotionally demanding contacts) displayed linear development, suggesting that the rate of growth remained constant over time, and eight dimensions had more complex macro trends (figure 1).

In one case (work postures), the trajectory was quadratic, ie, it first decelerated and then accelerated over time. In another case (workload), the trajectory was instead cubic (S-shaped), with one peak and one trough. However, the remaining working conditions displayed even more complex macro trends with several simultaneous trends. While psychological pressure and appreciation displayed quadratic and cubic trends, decision authority: when; overtime; time pressure; and bend and twist displayed a combination of linear, cubic, and quadratic trends.

The macro trends were particularly salient among the various aspects of job demands (table 4 and figure 1). Workload showed a clearly positive development (standardized change, 0.47), with a slight decline in later years. Overtime and time pressure were S-shaped with no clear direction over time. These quantitative demands were thus fluctuating over time. Difficulty of work tasks improved linearly (standardized change, 0.19), meaning less difficult work. Psychological pressure first clearly improved (standardized change, 0.49), but then slightly declined. A favorable development took place in the physical job demands, with work postures (standardized change, 0.16) and bend and twist (standardized change, 0.20) displaying mainly positive development after initial deterioration. Emotionally demanding contacts displayed linear negative development (standardized change, –0.08), suggesting slight deterioration in emotional demands.

Only two of eight job resources displayed a significant macro development trend. Decision authority over when to do work displayed S-shaped development with no clear direction over time, while appreciation (from workmates, patients, and/or clients) also displayed S-shaped development but with an improvement in recent years (standardized change, 0.16).

### Meso trends in development of job demands and resources

Significant variation in slopes was found for 15 of the 21 working conditions investigated (table 5). The analyses showed that the occupations developed differently over time for most working conditions, revealing substantial occupational trends, ie, meso trends, within the labor market. Among the job demands, the following 8 (of 13) conditions displayed significant variation in development across occupations (the variations in slope calculated as the low and high standardized change are given after each dimension): concentration (low=–0.90, high=0.66); psychological pressure (low=–0.11, high=0.88); work–leisure spillover (low=–0.47, high=0.37); time pressure (low=–0.28, high=0.76); emotionally demanding contacts (low=–0.33, high=0.18); violence and threats (low=–0.31, high=0.34); work postures (low=–0.11, high=0.44); and bend and twist (low=–0.01, high=0.40). The following demand dimensions did not display any significant meso trends: difficulty of work tasks, monotony, workload, overtime, and physical workload.

The meso trends were noticeable among job resources, with all except one resource dimension (social support from colleagues) displaying a significant meso trend. The following seven (out of eight) dimensions displayed significant variation in development across occupations (the variations in slope calculated as the low and high standardized change are given after each dimension): decision authority: pace (low=–0.58, high=0.42); decision authority: when (low=–0.33, high=0.37); decision authority: what, how (low=–0.31, high=0.38); unbound and free (low=–0.58, high=0.60); appreciation (low=–0.43, high=0.74); pause opportunities (low=–0.59, high=0.51); meaningfulness (low=–0.37, high=0.41).

**Figure 2:**
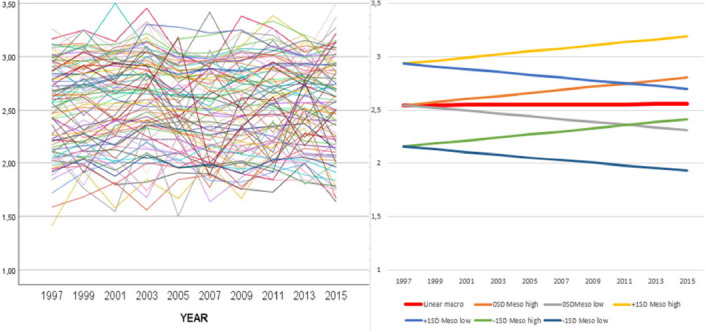
Example of meso-level observations of the variable “decision authority – when”. (Question: Are you able to determine when various work duties are to be carried out (for example, by choosing to work a bit faster on some days and taking it easier on other days)? Response alternatives: 1 = no, not at all, 2 = mostly not, 3 = mostly, 4 = always. Left panel: Aggregated observed data for all occupations, 1997–2015. Right panel: Meso trends shown as estimated trajectories (low and high) for selected starting points (intercepts) at –1 SD, 0 SD, and +1 SD. Estimated linear macro trend shown as a bold line..

An illustration of what the meso-level observations for occupations look like for the job resource Decision authority; when is presented in figure 2. The left-hand panel shows the observed data for occupations. The right-hand panel shows the low and high estimated trajectories for selected starting points, i.e., an occupation that starts at a certain point is estimated to decrease or increase within the lines shown.

### Polarization trends in development of job demands and resources

The random intercept was significant in all the working conditions measured, showing that different occupations had different “starting points” for their occupational trajectories of working conditions within the study period (table 4).

To detect a trend towards polarization, the covariances between the intercepts and slopes in the multilevel models were inspected. All covariances were either not significantly different from zero or were significantly negative; no covariances were significantly positive. Thus, no support was found for polarization trends in working conditions.

## Discussion

The main findings were a stable overall level of working conditions across occupations, divergent developments at the occupational level, and no conclusive support for a polarization trend. This study supported neither the ongoing upgrading nor polarization of work environments and working conditions in the occupational structure. The most important finding is that macro-level trends comprise a large variety of heterogeneous meso trends across occupational groups.

Accounts of an increase in stressful work environments are common in Swedish public debate (36, 37). The current study thus points to a more complicated picture. Its findings reveal that no clear improvement trend has occurred for most job demands and resources. This could mean that the deterioration in working conditions, with substantial increases in job demands as well as decreases in job control, that took place in Sweden in the 1990s is still present (eg, 6, 9, 14, 15.). But foremost, it discloses great heterogeneity of development between occupations. Consequently, experiences of change in the Swedish work environment vary greatly between workers in different occupational groups.

### Overall development of job demands and resources

At the macro level, most indicators of job demands and resources displayed no clear improvement trend. Concerning job demands, 13 indicators were included in the analysis and significant macro trends were evident in eight of them. Five displayed a positive trend, one a negative trend, and two nonlinear trends. Workload was the only quantitative demand that displayed a clear improvement trend. Two of four cognitive demands (difficulty of work tasks and psychological pressure) displayed deteriorating trends of lower levels, while two of three physical job demands (work postures and bend and twist) displayed improving trends. Only emotionally demanding contacts displayed a clear negative trend. On the resource side, only one job resource displayed a significant change at the macro-level, with increasing levels of appreciation from workmates, patients, and/or clients. The remaining 12 indicators of job demands, and resources displayed no clear macro-level changes between 1997–2015.

Thus, at the macro level, no radical changes in the working conditions of occupations were found over time; most working conditions remained fairly stable. This result both corresponds to (38, 39), and contrasts findings from other countries, where both positive (6, 39, 40) and negative (6, 40–44) overall trends have been observed, depending on exposure and time period. Concerning the trends detected, the study showed that job resources vary less over time at the occupational level than do job demands. This may indicate that job resources are more closely related to factors on other levels than the occupational, for example, the industry or workplace level (cf. 6).

### Variation across occupational groups

While the macro analysis of the work environment of occupations revealed few changes over time, the working conditions within different occupations were definitely changing. For all 21 included dimensions of working condition, the 89 included occupations had different starting points, and most of the dimensions displayed occupational variation in trajectories over time. Compared with the relatively modest changes at the macro level, these changes were quite substantial.

As illustrated in figure 2, the observed occupational developments in job demands and resources resemble a haystack with a jumble of development trends heading in different directions. While this haystack is difficult to interpret, the analytical framework presented here summarizes the occupational developments in a manageable number of statistical parameters. The most important conclusions of this analysis are that, first, for a few of the studied job demands and resources, the trends in all occupations are similar. Non-significant differences in slopes are only found for 6 of the 21 working conditions. Second, the differences in trends are in no cases unequivocally in the direction of improvement. However, there are cases in which the upper bound is clearly positive, while the lower bound is approaching zero, for example, in the cases of psychological pressure and bend and twist. Third, regarding some working conditions, the developments are strongly divergent between occupations (ie, the variations in coefficients), for example, in the cases of unbound and free, decision authority, concentration, and meaningfulness. This heterogeneity of trends may explain the non-trends in the above macro analysis results.

Previous studies have commonly looked for differences between subgroups based on, for example, gender, age, and level of education (see, for example 39, 40, 43–45,). The present findings suggest that it is equally important to consider differences in development between occupations and occupational groups (cf. 17, 41–42). The results support adding a labor market perspective taking the overall occupational structure into account.

Consequently, from an interventionist point of view, knowledge of how the job demands and resources vary between occupations that, for example, are female or male dominated, could add knowledge about contextual factors besides general gender differences (46). Policies on health and safety interventions might in this way be better targeted towards specific industries and occupations.

### Do work conditions develop in a polarized manner over time?

This study has revealed strong heterogeneity in the occupational trends of working condition indicators. However, when directly testing whether the job demands and resources for different occupations tend to diverge over time – a tendency that may indicate polarization of working conditions – no clear evidence was found. This is in line with earlier findings. For example, narrowing rather than increasing disparities in job security by occupational skill level were found for working Australian between 2000 and 2008 (39). Consequently, future research could attempt to identify other patterns. Furthermore, the distribution of individuals across these occupations needs to be introduced to determine how the workforce is distributed among occupations with different trends in working conditions. In addition, attention should be paid to labor market changes, such as employment conditions, that could explain these diverging development trends (cf. 47).

### Strengths and limitations

The main strength of this study is its representative sample of the Swedish labor market and working conditions over a fairly long period that has rarely been studied, despite changes in occupational health and safety conditions. A limitation is the decreasing response rates on the SWES along with a lower response rate among men, employees with low education, low income, and foreign background as well as among those with contract or part-time employment or with own businesses (30). Since several of these groups are concentrated in occupations at the “lowest end” of the occupational structure, – for example, in the platform or gig economy – employees with atypical employment and possibly more unregulated working conditions might not be captured to the same extent in the data. If this means that entire occupations fall out of the analysis this in turn might contribute to overestimation of the positive development of working conditions. However, even though there are no official statistics available on differences in dropout between different occupations in SWES our own compilations (see supplementary table S1) shows that the 21 occupations that were omitted form the analysis due to too few respondents are represented in almost all of the main occupational groups at the Swedish labor market. Thus, since all the occupations are given the same weight in GCM analysis our judgment is that the exclusion of very small occupations will not bias the data to any troublesome extent.

The SWES relies on self-reported measures, so measurement problems could affect the levels of reported exposure; however, as the present interest is in development over time and differences between groups, the risks of wrong conclusions should be smaller (48).

A prerequisite to adopt aggregation to the occupational level is that the individual level reports contain information that could be attributed to the occupational level. Thus, the individual level ICC (1,1) values are important to judge if the aggregates contain reliable information. As stated in the results section, we found that roughly 2–39% of the variance in the 24 studied working conditions could be attributed to the occupational meso level, meaning that the remaining variance may thus be explained by aspects associated with workplace, employee-specific characteristics, and measurement error. We judged that ICC (1,1) values >5% constituted a low occupational-level variance, and therefore omitted three dimensions which fell below this limit. We conclude that not all working conditions are suitable to study at the occupational level. However, the range of variance in our 21 studied working conditions that could be attributed to the occupational meso level is still large. Similarly, to ICC (1,1) values reported by eg, Madsen et al (49), and as can be seen in table 3, the ICC is consistently high for the physical demands in our study which makes them particularly suitable for studying with our choice of method. On the other hand, the ICC values on the dimensions covering social support are the lowest in our study and additional social support dimensions were left out of the final analysis due to even lower ICC. This means that these types of working conditions are probably more closely knitted to a specific workplace, employee-specific characteristics which has also been found in other studies (eg, 6.). Most dimensions studied, however, fall in the middle of these extremes often with a mixture of lower and higher ICCs within the same domain. Aggregated level reliability is however determined not only by the individual level intraclass correlation ICC (1,1) but also by the number of responses in each occupation (50). Accordingly, we considered both these parameters when deciding which variables and occupations to include. We decided to exclude occupations with few observations (N<15 for more than 50% of survey rounds). This decision was based on a trade-off between occupation level aggregated variable reliability versus the importance of retaining representability to the labor market as a whole. In order to follow the trajectories in small occupations there is a need for sampling more employees in those occupations.

The chosen time period starts after the major negative restructurings of the work environment during the 1990s. If the series had started before the crises of that time, these restructurings would have been taken into account, and what here appear to be improvements in the work environment might instead be regarded as partial recoveries at the end of a longer period of deterioration. In this study, we wanted to consider a broad range of job demands and resources, but only a few of these were available in the pre-crisis waves of the SWES.

### Concluding remarks

Based on self-report surveys, fairly high overall stability was found in the physical and psychosocial working conditions at the macro level of the Swedish labor market. However, this macro-level stability hides highly heterogeneous patterns of change among different occupational groups. The study gives no clear-cut support for the occupational structure moving in either an upgrading or polarizing direction concerning job demands and resources.

The results emphasize that policy-makers, employer organizations, and other decision-makers aiming to improve the work environment of employees need to take the contexts of industries, sectors, and occupations into account to have adequate information. We see tendencies toward such a contextual approach on the European level. Eurofound has defined different types of jobs based on job quality and identified how these are distributed across different industries on the European labor markets (51). The findings of our study clearly support this endeavor as a way forward for bodies that generate work environment statistics.

### Funding

The study was funded by the project “The Challenges of Polarization on the Swedish Labour Market” (Forte Dnr: 2016-07204).

### Ethics standards

The regional ethics committee in Gothenburg (Dnr 962-17) approved this study.

### Competing interests

The authors declare no competing interests.

## Supplementary material

Supplementary material
