# Supplementary material for: Occupational trajectories of working conditions in Sweden: Development trends in the workforce, 1997–2015
Source: Scand J Work Environ Health. 2021 Jun 29;47(5):335–48. doi: 10.5271/sjweh.3955 (PMC8259708; doi:10.5271/sjweh.3955)
Supplement: Supplementary material [file SJWEH-47-335-S001.pdf]

Occupational trajectories of working conditions in Sweden: Development trends in the workforce, 1997–2015

by Linda Corin, PhD,[1, 2] Anders Pousette, PhD,[3] Tomas Berglund, PhD,[2] Lotta Dellve, PhD,[2] Gunnel Hensing, PhD,[4] Lisa Björk, PhD [1, 2]

1. *Supplementary material*

2. *Correspondence to: Linda Corin, Institute of Stress Medicine, Region Västra Götaland, 413 19 Gothenburg, Sweden. [E-mail:linda.corin@vgregion.se]*

Table S1. Included as well as excluded occupations and their number of respondents per year in our sample as well as comparations with the number of employed in each occupation at the Swedish labour market per year.

SSYK96	Occupation	1997	1999	2001	2003	2005	2007	2009	2011	2013	2015	Average proportion in sample	Average proportion labor market*
011	Armed forces	54	35	27	23	36	26	17	32	28	24	0.27%	0.33%
111	*Legislators and senior government officials*	*6*	*8*	*12*	*9*	*8*	*10*	*4*	*14*	*5*	*5*	*0.07%*	*0.06%*
112	*Senior officials of special-interest organisations*	*3*	*2*	*2*	*2*	*11*	*6*	*1*	*3*	*3*	*4*	*0.03%*	*0.03%*
121	Directors and chief executives	41	18	47	60	81	68	50	81	58	71	0.51%	0.56%
122	Production and operations managers	188	173	192	224	229	190	183	269	207	16	1.67%	1.75%
123	Other specialist managers	150	154	132	146	172	189	144	223	142	90	1.38%	1.74%
131	Managers of small enterprises	196	185	183	175	155	113	112	107	63	161	1.30%	1.85%
211	Physicists, chemists and related professionals	20	16	9	19	19	22	16	25	10	15	0.15%	0.18%
212	*Mathematicians and statisticians*	*2*	*5*	*5*	*8*	*9*	*9*	*3*	*10*	*7*	*5*	*0.06%*	*0.05%*
213	Computing professionals	219	266	292	245	296	277	253	376	258	240	2.43%	2.29%
214	Architects, engineers and related professionals	137	131	165	180	239	204	171	217	183	158	1.59%	1.84%
221	Life science professionals	23	32	36	32	41	31	23	47	39	17	0.29%	0.10%
222	Health professionals (except nursing)	129	123	128	144	154	135	109	152	92	101	1.13%	1.15%
223	Nursing and midwifery professionals	133	113	124	129	134	117	109	159	98	85	1.07%	0.89%

231	College, university and higher education teaching professionals	86	80	79	58	78	46	60	68	52	55	0.59%	0.86%
232	Secondary education teaching professionals	199	217	223	194	221	181	149	207	106	91	1.60%	1.47%
233	Primary education teaching professionals	331	273	265	286	291	205	204	251	209	205	2.25%	2.20%
234	Special education teaching professionals	41	34	41	44	52	50	44	41	27	0	0.37%	0.29%
235	Other teaching professionals	45	42	52	49	64	38	36	69	56	41	0.44%	0.41%
241	Business professionals	228	293	353	381	407	346	280	359	293	295	2.89%	2.49%
242	Legal professionals	45	37	45	41	64	40	36	75	44	48	0.42%	0.43%
243	Archivists, librarians and related information professionals	25	22	29	40	42	25	24	45	22	15	0.26%	0.23%
244	Social science and linguistics professionals	13	15	26	33	25	16	23	17	19	22	0.19%	0.12%
245	Writers and creative or performing artists	151	157	146	138	194	152	112	176	94	103	1.27%	0.97%
246	*Religious professionals*	*10*	*5*	*7*	*13*	*12*	*15*	*14*	*14*	*7*	*8*	*0.09%*	*0.09%*
247	Public service administrative professionals	86	72	61	84	66	73	50	101	57	0	0.65%	1.20%
248	Administrative professionals of special-interest organisations	37	27	20	20	31	17	15	19	17	79	0.25%	0.19%
249	Psychologists, social work and related professionals	114	101	119	101	151	108	93	134	99	99	1.00%	0.86%
311	Physical and engineering science technicians	587	509	496	371	397	337	334	398	283	266	3.55%	3.28%
312	Computer associate professionals	50	117	134	128	148	109	96	123	91	76	0.96%	1.06%
313	Optical and electronic equipment operators	47	47	72	37	46	33	35	67	43	21	0.40%	0.22%
314	Ship and aircraft controllers and technicians	31	30	41	34	44	28	18	38	24	23	0.28%	0.17%
315	Safety and quality inspectors	23	16	25	27	29	21	25	40	28	16	0.22%	0.21%
321	*Agronomy and forestry technicians*	*8*	*8*	*10*	*12*	*14*	*7*	*5*	*11*	*14*	*17*	*0.09%*	*0.08%*
322	Health associate professionals (except nursing)	126	124	132	123	156	129	122	141	89	123	1.13%	0.91%
323	Nursing associate professionals	189	228	209	199	214	180	127	217	135	116	1.62%	1.75%
324	Life science technicians	37	36	39	57	38	22	24	25	10	12	0.27%	0.25%
331	Pre-primary education teaching associate professionals	271	290	256	243	301	232	202	313	190	189	2.22%	2.11%
332	Other teaching associate professionals	22	23	23	30	23	19	27	24	22	47	0.23%	0.16%
341	Finance and sales associate professionals	597	567	592	620	678	495	468	637	447	315	4.84%	4.56%

342	Business services agents and trade brokers	60	65	76	49	79	64	62	80	53	62	0.58%	0.50%
343	Administrative associate professionals	209	214	215	275	251	211	222	305	234	114	2.01%	1.80%
344	Customs, tax and related government associate professionals	66	69	56	59	75	45	36	61	39	72	0.52%	0.53%
345	Police officers and detectives	46	64	59	38	41	49	42	62	46	45	0.44%	0.42%
346	Social work associate professionals	100	93	100	94	133	78	76	104	74	62	0.82%	0.83%
347	Artistic, entertainment and sports associate professionals	77	84	72	63	85	63	60	92	76	115	0.70%	0.37%
348	*Religious associate professionals*	*4*	*2*	*8*	*5*	*1*	*4*	*2*	*3*	*1*	*3*	*0.03%*	*0.03%*
411	Office secretaries and data entry operators	217	179	153	142	119	82	85	102	51	32	1.04%	1.01%
412	Numerical clerks	151	158	202	218	215	188	147	189	119	136	1.54%	1.46%
413	Stores and transport clerks	237	271	251	233	229	204	151	149	84	114	1.72%	1.72%
414	Library and filing clerks	38	25	20	15	22	26	11	14	9	12	0.17%	0.13%
415	Mail carriers and sorting clerks	100	85	98	75	78	62	47	48	36	16	0.58%	0.61%
419	Other office clerks	442	367	322	232	305	192	156	147	126	181	2.21%	2.35%
421	Cashiers, tellers and related clerks	112	108	133	86	128	78	68	94	50	71	0.83%	0.62%
422	Client information clerks	198	173	191	165	158	149	110	119	97	55	1.26%	1.17%
511	Travel attendants and related workers	14	20	23	18	27	26	10	19	18	12	0.17%	0.17%
512	Housekeeping and restaurant services workers	179	178	172	185	191	186	141	166	97	107	1.43%	1.40%
513	Personal care and related workers	1352	1365	1435	1432	1492	1149	997	1374	827	662	10.79%	12.84%
514	Other personal services workers	72	78	71	59	72	74	69	83	52	56	0.61%	0.27%
515	Protective services workers	84	97	75	85	68	71	63	78	62	37	0.64%	0.81%
521	*Fashion and other models*	*0*	*0*	*0*	*0*	*0*	*0*	*0*	*0*	*0*	*0*	*0.00%*	*0.00%*
522	Shop and stall salespersons and demonstrators	510	502	593	573	675	494	450	557	334	381	4.53%	4.94%
611	Market gardeners and crop growers	113	67	59	80	89	46	49	54	29	39	0.56%	0.40%
612	Animal producers and related workers	89	84	48	54	60	42	37	55	24	10	0.45%	0.19%
613	Crop and animal producers	35	94	131	106	78	63	49	53	35	35	0.61%	0.11%
614	Forestry and related workers	30	35	41	35	29	21	21	22	10	19	0.23%	0.10%
615	*Fishery workers, hunters and trappers*	*5*	*7*	*10*	*7*	*4*	*10*	*5*	*10*	*7*	*14*	*0.07%*	*0.01%*
711	*Miners, shotfirers, stone cutters and carvers*	*13*	*14*	*10*	*12*	*14*	*9*	*9*	*12*	*8*	*6*	*0.10%*	*0.08%*

712	Building frame and related trades workers	302	282	327	328	352	309	273	312	211	144	2.54%	2.33%
713	Building finishers and related trades workers	346	348	307	309	310	246	219	344	187	222	2.53%	2.37%
714	Painters, building structure cleaners and related trades workers	119	87	69	74	98	63	51	81	46	40	0.65%	0.55%
721	Metal moulders, welders, sheet-metal workers, structural-metal preparers and related workers	156	106	116	111	120	103	102	123	56	43	0.93%	0.85%
722	Blacksmiths, toolmakers and related trades workers	82	69	68	64	64	36	33	37	23	43	0.46%	0.35%
723	Machinery mechanics and fitters	210	213	198	196	189	134	125	156	110	84	1.44%	1.36%
724	Electrical and electronic equipment mechanics and fitters	142	107	144	163	176	124	94	163	78	83	1.14%	0.82%
731	Precision workers in metal and related materials	31	27	29	27	21	28	15	20	11	13	0.20%	0.11%
732	*Potters, glassmakers and related trades workers*	*11*	*7*	*9*	*9*	*12*	*3*	*2*	*7*	*2*	*0*	*0.06%*	*0.04%*
733	*Handicraft workers in wood, textile, leather and related materials*	*7*	*5*	*3*	*3*	*5*	*2*	*3*	*2*	*6*	*0*	*0.04%*	*0.00%*
734	*Craft printing and related trades workers*	*25*	*17*	*34*	*24*	*26*	*11*	*7*	*8*	*6*	*3*	*0.14%*	*0.16%*
741	Food processing and related trades workers	52	61	54	48	43	41	12	25	23	14	0.33%	0.23%
742	Wood treaters, cabinetmakers and related trades workers	16	13	20	17	16	15	3	5	10	31	0.13%	0.06%
743	Garment and related trades workers	49	23	19	17	21	11	9	16	7	5	0.16%	0.07%
744	*Pelt, leather and shoemaking trades workers*	*3*	*3*	*3*	*2*	*3*	*5*	*2*	*1*	*3*	*2*	*0.02%*	*0.01%*
811	*Mineral-processing-plant operators*	*6*	*5*	*3*	*10*	*4*	*2*	*8*	*5*	*6*	*6*	*0.05%*	*0.04%*
812	Metal-processing-plant operators**	30	39	29	41	35	23	10	26	7	14	0.23%	0.39%
813	Glass, ceramics and related plant operators**	10	6	6	3	6	9	10	5	1	0	0.06%	0.04%
814	Wood-processing- and papermaking-plant operators**	86	63	63	61	85	47	38	46	25	26	0.48%	0.51%
815	Chemical-processing-plant operators**	14	16	11	17	12	12	10	6	7	9	0.10%	0.17%
816	Power-production and related plant operators	28	24	32	24	30	21	28	24	15	16	0.22%	0.19%
817	*Industrial-robot operators*	*15*	*3*	*9*	*4*	*12*	*5*	*2*	*2*	*4*	*4*	*0.05%*	*0.02%*
821	Metal- and mineral-products machine operators	187	219	183	176	190	153	81	130	78	71	1.31%	1.24%
822	Chemical-products machine operators	30	44	49	39	35	35	25	32	23	16	0.29%	0.32%

823	Rubber- and plastic-products machine operators	53	42	43	49	48	35	26	19	24	12	0.31%	0.35%
824	Wood-products machine operators	36	36	34	31	32	28	21	22	13	11	0.24%	0.39%
825	Printing-, binding- and paper-products machine operators	89	71	65	47	47	31	32	37	18	14	0.40%	0.33%
826	Textile-, fur- and leather-products machine operators	46	34	32	27	26	18	11	24	11	8	0.21%	0.17%
827	Food and related products machine operators	54	61	75	64	51	54	29	50	19	25	0.43%	0.55%
828	Assemblers	217	265	219	192	172	159	88	152	94	33	1.42%	1.36%
829	Other machine operators and assemblers	26	49	41	33	30	24	19	33	10	37	0.27%	0.74%
831	*Locomotive-engine drivers and related worker*	*17*	*15*	*8*	*15*	*18*	*14*	*12*	*21*	*9*	*11*	*0.13%*	*0.14%*
832	Motor-vehicle drivers	319	289	339	298	354	257	246	314	204	170	2.49%	2.47%
833	Agricultural and other mobile-plant operators	149	155	189	162	198	158	148	221	165	93	1.46%	0.82%
834	*Ships' deck crews and related workers*	*8*	*6*	*2*	*4*	*9*	*2*	*2*	*3*	*1*	*1*	*0.03%*	*0.05%*
911	*Street vendors and market salespersons*	*2*	*1*	*3*	*1*	*3*	*4*	*1*	*1*	*1*	*2*	*0.02%*	*0.00%*
912	Helpers and cleaners	268	240	239	208	233	148	145	202	126	95	1.70%	1.80%
913	Helpers in restaurants	173	181	156	137	139	118	70	130	76	61	1.11%	1.60%
914	Doorkeepers, newspaper and package deliverers and related workers	87	58	75	69	82	44	47	46	25	37	0.51%	0.42%
915	Garbage collectors and related labourers	23	24	22	24	16	6	12	18	10	9	0.15%	0.24%
919	*Other sales and services elementary occupations*	*6*	*17*	*14*	*10*	*10*	*15*	*8*	*12*	*5*	*10*	*0.10%*	*0.67%*
921	*Agricultural, fishery and related labourers*	*11*	*8*	*2*	*2*	*5*	*6*	*4*	*8*	*6*	*4*	*0.05%*	*0.07%*
931	*Mining and construction labourers*	*4*	*2*	*3*	*0*	*1*	*2*	*0*	*1*	*0*	*3*	*0.02%*	*0.09%*
932	Manufacturing labourers	42	38	45	42	86	83	53	65	36	22	0.46%	0.88%
933	Transport labourers and freight handlers	46	27	14	19	25	20	23	35	43	47	0.27%	0.35%
**Total**		**12886**	**12535**	**12878**	**12355**	**13538**	**10671**	**9152**	**12367**	**8110**	**7336**		

Note.

* We have calculated the proportion of respondents in each occupation in our sample as well as the proportion of respondents in each occupation at the Swedish labour market to facilitate comparations. However. the numbers are not exactly comparable since the statistics covering the whole labor market is only available for the years 2001-2013.

**Occupations that have been merged into one in the analyses. new occupational code 810.

Italics Occupations that have been omitted from the analyses due to a very small number of respondents is in italics.
